# A novel crosstalk between TLR4- and NOD2-mediated signaling in the regulation of intestinal inflammation

**DOI:** 10.1038/srep12018

**Published:** 2015-07-08

**Authors:** Hajeong Kim, Quanju Zhao, Hua Zheng, Xin Li, Tuo Zhang, Xiaojing Ma

**Affiliations:** 1 State Key Laboratory of Microbial Metabolism, Sheng Yushou Center of Cell Biology and Immunology and School of Life Sciences & Biotechnology, Shanghai Jiao Tong University, 800 Dongchuan Road, Shanghai, China 200240; 2 Department of Microbiology and Immunology, Weill Cornell Medical College, 1300 York Avenue, New York, NY, USA 10065; 3 Department of Breast Surgery, Xiangya Hospital, Central South University, 87 Xiangya Road, Changsha, Hunan, China 410008

## Abstract

Although Toll-like receptor 4 (TLR4)- and nucleotide-binding oligomerization domain 2 (NOD2)-mediated signaling mechanisms have been extensively studied individually, the crosstalk between them in the regulation of intestinal mucosal defense and tissue homeostasis has been underappreciated. Here, we uncover some novel activities of NOD2 by gene expression profiling revealing the global nature of the cross-regulation between TLR4- and NOD2-mediated signaling. Specifically, NOD2 is able to sense the intensity of TLR4-mediated signaling, resulting in either synergistic stimulation of Interluekin-12 (IL-12) production when the TLR signaling intensity is low; or in the inhibition of IL-12 synthesis and maintenance of intestinal mucosal homeostasis when the TLR signaling intensifies. This balancing act is mediated through receptor-interacting serine/threonine kinase 2, and the transcriptional regulator CCAAT/enhancer-binding protein α (C/EBPα) via its serine 248 phosphorylation by Protein Kinase C. Mice deficient in C/EBPα in the hematopoietic compartment are highly susceptible to chemically induced experimental colitis in an IL-12-dependent manner. Additionally, in contrast to the dogma, we find that the major Crohn’s disease-associated NOD2 mutations could cause a primarily immunodeficient phenotype by selectively impairing TLR4-mediated IL-12 production and host defense. To restore the impaired homeostasis would be a way forward to developing novel therapeutic strategies for inflammatory bowel diseases.

Inflammatory bowel disease (IBD), particularly Crohn’s disease (CD), involves the interplay of commensal and pathogenic bacteria, genetic mutations, and immunoregulatory defects in both innate and adaptive immune systems^1^. CD has a strong genetic basis^2^,^3^. Nucleotide-binding oligomerization domain 2 (NOD2) is an important regulator in the broad context of host resistance to microbial challenge as well as maintenance of tissue homeostasis. The gene encoding NOD2, *CARD15*, is the first susceptibility gene discovered for CD^2^,^3^. Three main variants of NOD2 have been identified: R702W, G908R, and 1007fs, which together represent ~80% of the total NOD2 mutations independently associated with susceptibility to CD^4^,^5^. How these human NOD2 mutants contribute to the development and pathogenesis of CD has been quite controversial, reflecting the complexity of the physio-biological properties of NOD2^6^,^7^,^8^,^9^.

Although there is documented evidence for a synergism between TLR2/4- and NOD2-mediated signaling in cytokine production^10^,^11^,^12^, NOD2 is able to inhibit TLR2/4-mediated induction of inflammatory cytokine production and induce immune tolerance and homeostasis^7^,^13^,^14^. Yet, the mechanisms whereby NOD2 confers these activities, particularly in immune cells, remain highly murky. In mice, Watanabe *et al.* showed that intact NOD2 signaling inhibited TLR2-driven activation of NF-κB, principally, c-Rel^7^. NOD2 deficiency or the presence of a CD-like mutation in NOD2 increased TLR2-mediated activation of c-Rel, and Th1 responses were enhanced^7^.

The critical roles of IL-12 and IL-23 in human CD pathogenesis have been strongly implicated in human clinical studies demonstrating that CD but not ulcerative colitis is associated with high levels of both IL-12 and IL-23 secretion^15^,^16^, and blocking p40 by monoclonal antibodies is therapeutically beneficial^17^,^18^. However, because IL-23 shares the p40 subunit with IL-12, the role of IL-12 had not been precisely determined in early studies using neutralizing p40 Abs. Becker *et al.* demonstrated that IL-23p19-deficient mice were highly susceptible to the development of trinitrobenzene sulfonic acid (TNBS)-induced colitis and exhibited more severe colitis than wild type (WT) mice. Further analyses revealed that dendritic cells (DCs) from p19-deficient mice produced elevated levels of IL-12, and that IL-23 down-regulated IL-12 expression upon TLR ligation. Additionally, *in vivo* blockade of IL-12p40 in IL-23-deficient mice rescued mice from lethal colitis. This study clearly reveals a cross-regulation of IL-12 expression by IL-23 as a key regulatory pathway during initiation of T cell dependent colitis^19^. Strober *et al.* showed that NOD2 activation by its ligand muramyl dipeptide (MDP), a conserved motif present in peptidylglycan (PGN) from both Gram-positive and Gram-negative bacteria^20^,^21^, could downregulate responses to TLR stimulation, and thus murine cells lacking NOD2 mount increased responses to such stimulation^22^. Therefore, interactions between NOD2 and specific TLR pathways represent important but understudied modulatory mechanisms of innate and adaptive responses, particularly in the context of intestinal inflammatory diseases. The current study was undertaken to further investigate this novel but overlooked aspect of immunoregulation at multiple mechanistic levels.

## Results

### NOD2-mediated signaling interacts with TLR4-mediated signaling

**Figure 1:**
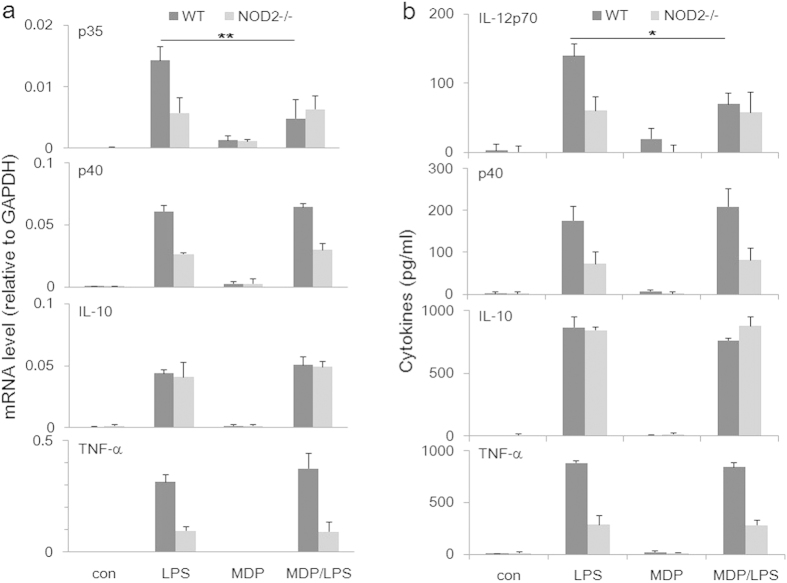
MDP-independent and dependent activities of NOD2. Bone marrow macrophages were derived with rM-CSF from WT and NOD2-KO mice, stimulated with LPS (500 ng/ml) with or without MDP (5 μg/ml), followed by determination of expressed mRNA and secreted cytokine levels by real time PCR (**a**) and ELISA (**b**), respectively. Data represent means of three trials with SE. *p < 0.05; **p < 0.01.

To assess the role of NOD2 in TLR4-mediated production of important cytokines, we derived bone marrow macrophages (BMDMs) from WT and NOD2-knockout (KO) mice, stimulated them with LPS with or without MDP, followed by analyzing expressed cytokine mRNA and secreted protein levels. As shown in Fig. 1a (mRNA) and **b** (protein), NOD2 deficiency strongly reduced LPS-induced levels of IL-12p40 (shared subunit by IL-12 and IL-23), IL-12p70 and TNF-α, but not that of IL-10. MDP by itself didn’t induce detectable levels of these cytokines. The combination of LPS and MDP reduced the level of p35 mRNA, and accordingly the level of IL-12 in a selective manner because none of the other cytokines were affected by the MDP treatment. These data suggest that endogenous NOD2 is required for LPS-induced production of inflammatory cytokines in an MDP-independent manner. In contrast, when activated by MDP, NOD2 acts as a selective inhibitor of IL-12p35 gene transcription, illustrating the crosstalk between TLR4- and MDP-induced signaling that leads to a highly selective control of IL-12 production.

**Figure 2:**
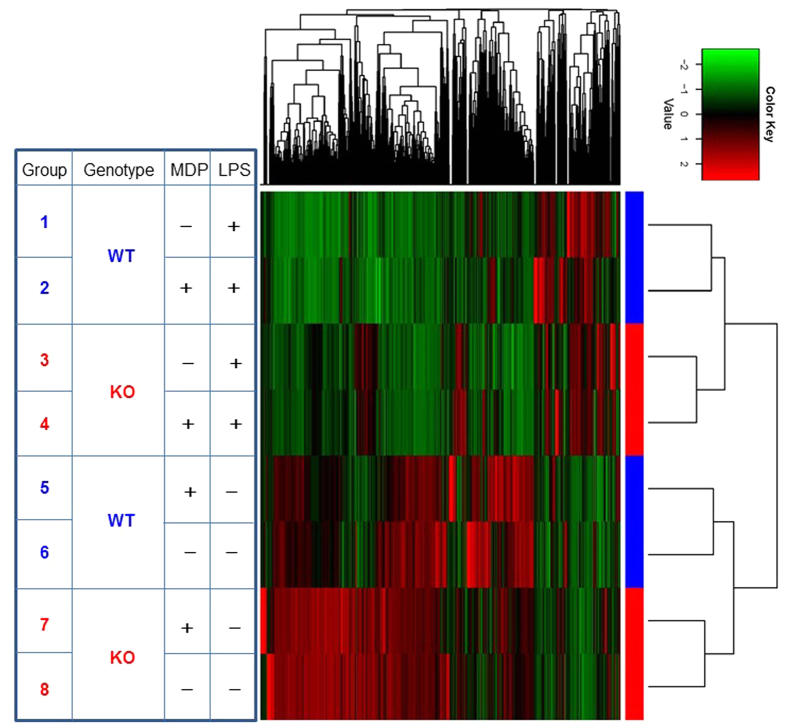
Clustering analysis of RNAseq profiling in WT vs NOD2 KO macrophages. RNA samples from WT and NOD2 KO macrophages treated with MDP or LPS or both were subjected to NexGen RNA-sequencing (whole exon). Genes of low expressions (RPKM <0.0) were filtered out. The RPKM values were further normalized per gene over all samples, to be specific, for each gene the mean and standard deviation (stddev) of RPKM over all samples were calculated, and the RPKM was linearly transformed using the formula (rpkm-mean)/stddev. The heatmap was then generated by heatmap.2 in the R gplots package. By default, heatmap.2 uses Euclidean measure to obtain distance matrix and complete agglomeration method for clustering.

For a more comprehensive understanding of the interaction between NOD2- and TLR4-mediated signaling, we performed whole exon NexGen RNA sequencing of macrophages from WT and NOD2 KO mice treated with LPS alone or with both LPS and MDP together. Clustering analysis of the sequencing data involving 17484 genes (Fig. 2) shows that in WT macrophages, MDP and LPS stimulated different sets of genes (compare Groups 5 and 1 against Group 6). MDP treatment inhibited many of a gene induced by LPS while also stimulated the expression of numerous others (compare Group 2 against Group 1). Strikingly, in the absence of NOD2, macrophages were spontaneously and broadly activated, which were strongly suppressed by LPS treatment (compare Group 3 to Group 8), again demonstrating a mutually restrictive relationship between the two signaling pathways.

More detailed analyses revealed that with LPS alone, only one gene was differentially upregulated more than 6-fold in mRNA expression in LPS-activated WT macrophages over NOD2-deficient cells: growth differentiation factor 15 (GDF15) (Table 1). Under the condition of MDP and LPS, only five genes were differentially upregulated more than 6-fold compared to NOD2-deficient cells, including also GDF15 (Table 2). In contrast, under both of these conditions in the absence of NOD2, large numbers of genes were upregulated, many dramatically. These “asymmetric” expression patterns suggest that NOD2 signaling primarily functions to limit, rather than to augment, LPS-induced inflammatory activities.

### NOD2-and TLR4-mediated signaling mutually antagonizes on IL-12p35 gene transcription

**Figure 3:**
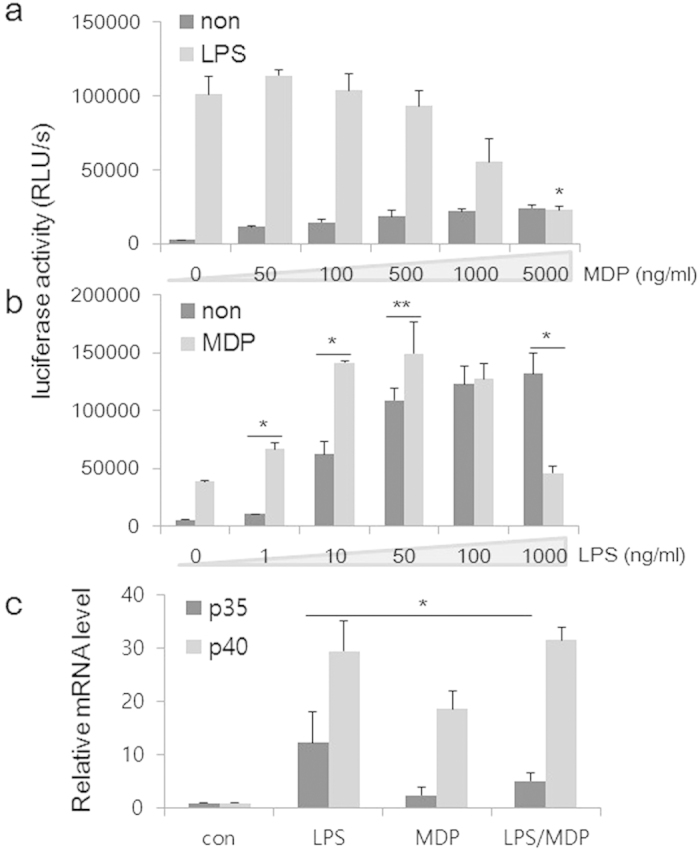
Reciprocal regulation of LPS- and NOD2-mediated signaling in IL-12 transcription. An IL-12p35 promoter-reporter construct was transfected into RAW264.7 cells. Twenty-four hours later, cells were stimulated for 6 h with LPS (500 ng/ml) in the presence of varying amounts of MDP as indicated (**a**), or with MDP (5 μg/ml) in the presence of increasing LPS concentrations (**b**). Cells were then harvested and cell lysate prepared. Luciferase activity was measured. Data represents mean plus SD of three separate experiments. (**c**) Peripheral blood-derived primary human monocytes obtained from normal donors were stimulated with LPS (500 ng/ml) for 6 h in the presence or absence of MDP (5 μg/ml). Total RNA was isolated and analyzed by real time quantitative PCR, and normalized against GAPDH. Data are expressed as relative levels to that of the unstimulated cells, which was set as 1. Data represent mean plus SEM of three donors.

To further characterize the selective targeting of the interaction between TLR4- and NOD2-signaling on IL-12 gene expression at the transcriptional level, we used a well-established reporter system in which the human IL12p35 gene promoter from −1082 to +61 linked to the firefly luciferase gene^23^ was transiently transfected into the mouse macrophage cell line RAW264.7. We analyzed the transcription of IL-12p35 promoter-luciferase reporter gene under these conditions. As shown in Fig. 3a, LPS induced p35 transcriptional activity was inhibited by MDP in a dose-dependent manner. Reciprocally, MDP enhanced p35 promoter activity at low doses of LPS (1–50 ng/ml), whereas it inhibited the transcription at the high dose (1 μg/ml) (Fig. 3b). The selective inhibition of MDP on high doses of LPS-induced transcription of the p35 but not p40 gene was corroborated on the endogenous counterparts in primary human monocytes (Fig. 3c). Thus, NOD2 signaling can “sense” the intensity of TLR4-induced cellular activation, and plays a dual role in the production of IL-12.

### NOD2-mediated regulation of IL-12 expression is RIP2-dependent and NF-κB-independent

**Figure 4:**
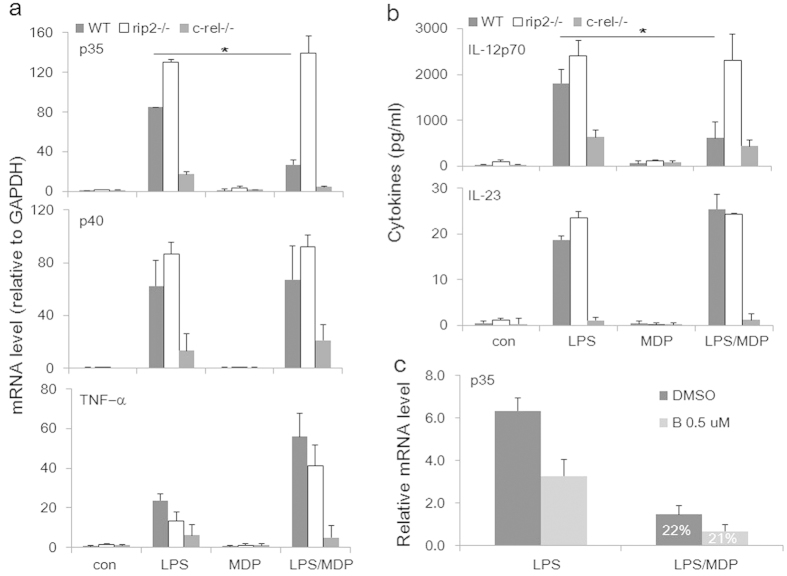
RIP2 and c-Rel in NOD2-mediated regulation of IL-12p35 and p40 expression. Thioglycolate-elicited peritoneal macrophages from wild-type (WT), Rip2^−/−^ and Crel^−/−^ mice were stimulated for 6 h with LPS (500 ng/ml) with or without MDP (5 μg/ml). RNA analyses were carried out by real time RT-PCR for (**a**) IL-12p35 (upper), p40 (middle), and TNF-α (lower). (**b**) Cytokines were measured from culture supernatant by ELISA for IL-12 (upper) and IL-23 (lower). (**c**) THP-1 cells were stimulated with LPS for 6 h with a low dose of Bay11–7082 (0.5 μM). Levels of p35 mRNA were analyzed by real-time RT-PCR. Numbers within the bars (22% and 21%, respectively) in MDP-treated cells indicate the percentages of remaining mRNA levels in the presence of Bay11–7082 compared to controls.

To further delineate the molecular mechanisms underlying NOD2-mediated selective inhibition of IL-12p35 gene transcription, we analyzed macrophages derived from mice deficient in receptor interacting protein 2 (RIP2)^24^, the protein kinase downstream of NOD2. We observed that in mice genetically deficient in RIP2, the inhibition of IL-12p35 mRNA expression by MDP in LPS-activated, highly inflammatory peritoneal macrophages was completely lost (Fig. 4a, upper panel, white bars), in comparison to the lack of responses of the p40 (mid-panel) and TNF-α (lower panel) genes in the same cells. The RIP2 dependency is consistent with the previous observation by Biswas *et al.* that RIP2- and NOD2-deficient mice share the same susceptibility to *Helicobacter hepaticus*-induced granulomatous inflammation of the ileum, characterized by an increased expression of Th1-related genes and inflammatory cytokines^25^.

In contrast, in mice deficient in c-Rel, the most vital component of the NF-κB complex for IL-12p35 transcription^26^, the relative degree of inhibition by MDP was intact in activated macrophages (Fig. 4a, upper panel, gray bars), although the absolute levels of p35 transcription were severely reduced. The responses of IL-12 and IL-23 protein secretion were very similar to those of the individual cytokine chains at mRNA levels (Fig. 4b). The NF-κB independency was corroborated by treating THP-1 cells (a human macrophage cell line) with a well-known and highly specific inhibitor of IκBα phosphorylation, Bay11–7082^27^, which at the low dose of 0.5 μM resulted in a partial inhibition of IL-12p35 mRNA synthesis (Fig. 4c), while having little impact on the relative degrees to which MDP inhibited p35 mRNA levels in activated cells without (black bars) or with (gray bars) the inhibitor.

### NOD2-and LPS-mediated regulation of IL-12p35 transcription via a proximal promoter element

**Figure 5:**
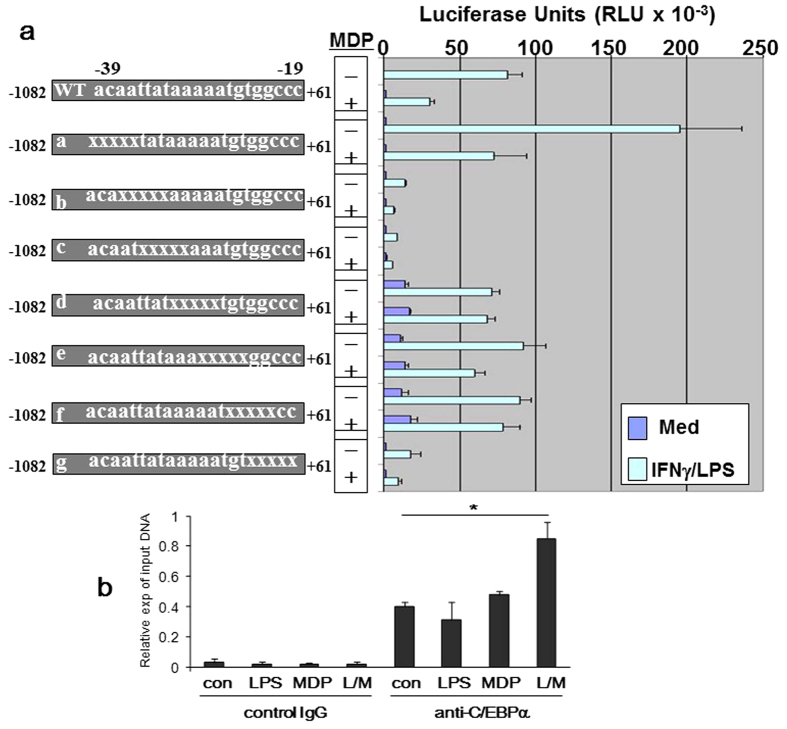
MDP regulates IL-12p35 transcription through a proximal promoter element. Various human IL-12p35 promoter-reporter base-substitution (transversions) constructs were transfected into RAW264.7 cells. Twenty-four hours later, cells were stimulated with LPS (500 ng/ml) in the presence of MDP (5 μg/ml) for 6 h. Cells were harvested and cell lysates prepared. Luciferase activity was measured. Data represents mean plus SD of three separate experiments.

To further explore the transcriptional mechanism whereby MDP through activation NOD2 regulates IL-12p35 gene expression, a series of 5′ and 3′ deletion constructs of the IL-12p35 promoter were tested to localize the *cis*-element, named “NOD2 Response Element” (N2RE), which mediated MDP-induced inhibition of p35 transcription. The systematic search led to a small region between −39 and −29 upstream of the transcription initiation site of the p35 promoter with the sequence TATAAAAATGTGGC (Fig. 5a). Base-substitutions with this region in reporter constructs b and c resulted in a dramatic loss of the transcriptional activity, presumably due to the disruption of the critically important TATA box^28^. Changes in constructs d, e, and f, however, resulted in elevated basal transcription levels while the LPS-stimulated transcriptional activity was not reduced compared to the wild type construct, and almost complete loss of the promoter’s response to MDP-induced inhibition.

An in silico analysis of the 14-bp NRE sequence critical for the MDP response using the transcriptional factor site prediction algorithm, Matlnspector^TM^ (Genomatix, Germany), unveiled a link between a member of the CCAAT/enhancer-binding protein (C/EBP) transcription factor family, C/EBPα, and the basal transcription machinery at the TATA box. C/EBPα is a key regulator of myeloid differentiation^29^,^30^. It is critical for early granulocytic differentiation via induction of granulocyte-specific genes^31^,^32^. Loss of CEBPα function in myeloid cells *in vitro* and *in vivo* leads to a differentiation block, similar to that observed in blasts from acute myeloid leukemia (AML) patients^33^,^34^. Nerlov *et al.* found that the three C/EBPα transactivation elements (TEs) synergistically activate transcription in mammalian cells; and two of these elements, TE-I and -II, co-operatively mediate *in vitro* binding of C/EBPα to TATA-box binding protein (TBP) and transcription factor IIB (TFIIB), two essential components of the RNA polymerase II basal transcriptional apparatus^35^.

By chromatin immunoprecipitation (ChIP) assay, we confirmed the physical interaction of C/EBPα with this IL-12p35 promoter element in bone marrow derived murine macrophages. As shown in Fig. 5b, C/EBPα binding was constitutively present and was increased significantly following dual treatment with LPS and MDP, but not by either agent alone, consistent with the functional outcome.

### Hematopoietic C/EBPα is essential in MDP-induced inhibition of IL-12 production

**Figure 6:**
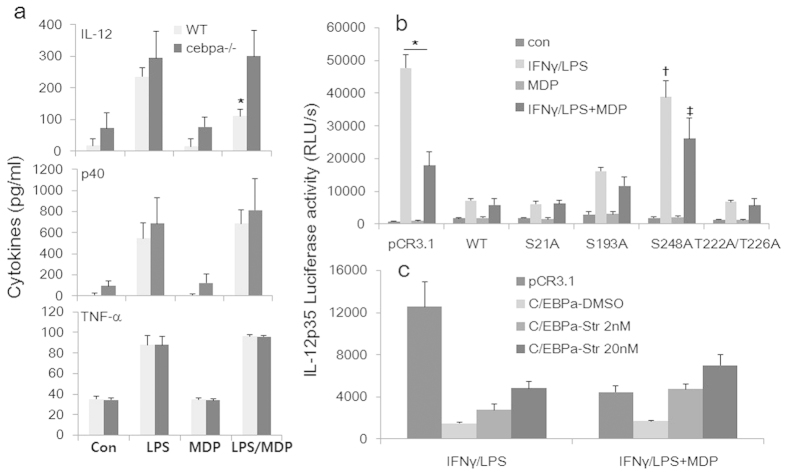
Role of C/EBPα and PKC in NOD2-mediated regulation of IL-12 expression. (**a**) Macrophages from WT and conditional C/EBPα-deficient mice were stimulated with LPS in the presence or absence of MDP, followed by cytokine measurements by ELISA for IL-12, p40, and TNF-α. (**b**) Transient cotransfections were carried out with the human IL-12p35 reporter and one of five C/EBPα expression vectors: WT, S21A, S193A, T222A/T226A, S248A phosphorylation mutants. Results represent three experiments. (**c**) Transient cotransfections were carried out as in (b). Various amounts of the general PKC inhibitor staurosporine (Str) were added to the culture as indicated.

To determine whether C/EBPα was critical to MDP-inhibition of IL-12 production, we isolated BMDMs from WT and conditional C/EBPα KO mice. Mx1-Cre transgene expression can be induced by administration of polyinosinic-polycytidylic acid [poly (I:C)], leading to deletion of the floxed *cebpa* gene in the hematopoietic compartment, as we described previously^36^. The BMDMs were stimulated with LPS with or without MDP, followed by cytokine measurements by ELISA. As shown in Fig. 6a, loss of C/EBPα did not affect LPS-induced IL-12p70, p40, and TNF-α production but caused a specific reversal of the inhibition of IL-12p70 production by MDP, confirming the essentiality of C/EBPα in this pathway.

Posttranslational modifications of C/EBPα, such as phosphorylation at different serine/threonine residues, have been found to be an important mechanism of regulating its transcriptional activity^37^,^38^. We investigated the functionality of the phosphorylation of C/EBPα using different serine/threonine phosphorylation mutants of C/EBPα^36^ cotransfected with the IL-12p35 luciferase reporter into RAW264.7 cells. As shown in Fig. 6b, among the four established C/EBPα mutants tested, only the S248A mutant of C/EBPα completely lost its inhibitory activity against p35 transcription whereas the other three mutants retained full repressive activity. Since protein kinase K (PKC) has been implicated in the phosphorylation of S248 in Ras-mediated signaling and activation of C/EBPα in granulocytic differentiation^39^, we sought to determine the role of PKC and S248 in the activity of C/EBPα using a potent pan-PKC inhibitor, staurosporine. The inhibition of PKC resulted dose-dependently but partially in the enhancement of the p35 transcriptional suppression caused by the overexpression of C/EBPα (Fig. 6c).

Taken together, the data clearly demonstrate that (1) C/EBPα is a physiological mediator of MDP-induced inhibition of p35 expression and IL-12 production in inflammatory macrophages; (2) C/EBPα’s activity is critically dependent on serine 248 of C/EBPα; (3) PKC activities are required for MDP to regulate p35 transcription in LPS activated macrophages; (4) C/EBPα acts in this pathway directly by binding to the p35 gene promoter at the N2RE, blocking its transcription.

### Hematopoietic C/EBPα mediates MDP’s protection against experimental colitis

**Figure 7:**
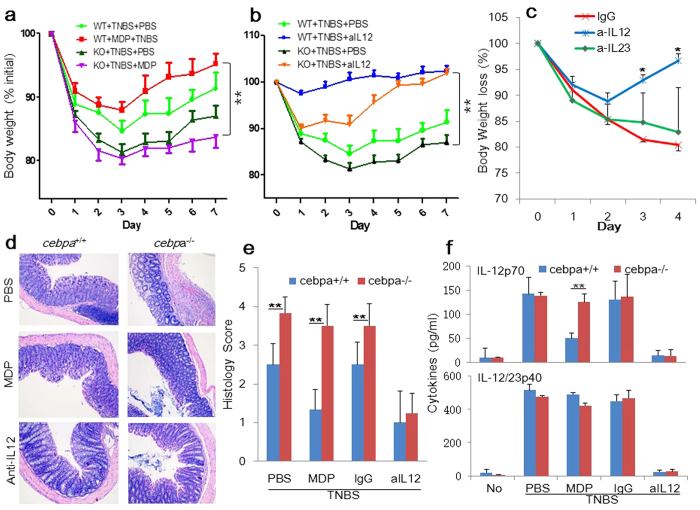
Role of C/EBPα and IL-12 in TNBS-induced colitis. (**a**) TNBS colitis was induced in WT and *cebp*α KO mice. MDP was administered i.p. on days –3, –2, and –1 (n = 9/group). Anti-IL12 antibody (**b**, **c**) or anti-IL-23 (**c**), or the control IgG were administrated on days 0, and 2 (n = 4/group). Mean body weight of three independent experiments with SE is represented. **(c)** H&E-staining of colonic tissues of NBS-, MDP- or anti-IL-12-treated mice harvested on day 4 are shown in 100x magnification showing massive infiltration of mononuclear cells as well as destruction of crypt architecture. (**d**) Histology scores of the colonic tissues harvested on day 4. **p < 0.01. (**f**) Sera from the mice were collected on day 4 and analyzed for cytokine production by ELISA (IL-12 and IL-23p40). **p < 0.01.

To determine definitively whether MDP’s rescuing effects on TNBS-induced colitis^13^ were dependent on C/EBPα, we used the conditional C/EBPα-deficient mice in which hematopoietic cell-specific deletion of the *cebpa* gene is initiated by the administration of poly I-C^36^. As shown in Fig. 7a, compared to WT mice, C/EBPα-deficient mice were more susceptible to colitis-associated weight loss. They also displayed more severe colonic tissue inflammation induced by TNBS administration (Fig. 7d,e). Crucially, MDP’s ability to protect mice from TNBS-induced colitis was completely lost in C/EBPα-deficient mice (Fig. 7a). Further, we tested the effect of neutralizing antibodies against IL-12 and IL-23, respectively. Compared with the control IgG-treated mice, colitis was completely rescued by the IL-12-neutralizing antibody in WT and C/EBPα-deficient mice (Fig. 7b) but not by anti-IL-23 (Fig. 7c), consistently with the degrees of serum levels of IL-12 (Fig. 7f).

Together, these data demonstrate that loss of hematopoietic C/EBPα renders greater susceptibility to TNBS-induced colitis; IL-12 is the primary target in the MDP/NOD2-C/EBPα regulatory axis; MDP requires C/EBPα to selectively inhibit IL-12 production and to resist TNBS-induced pathogenesis.

### 1007fs acts as a “dominant negative” against NOD2

**Figure 8:**
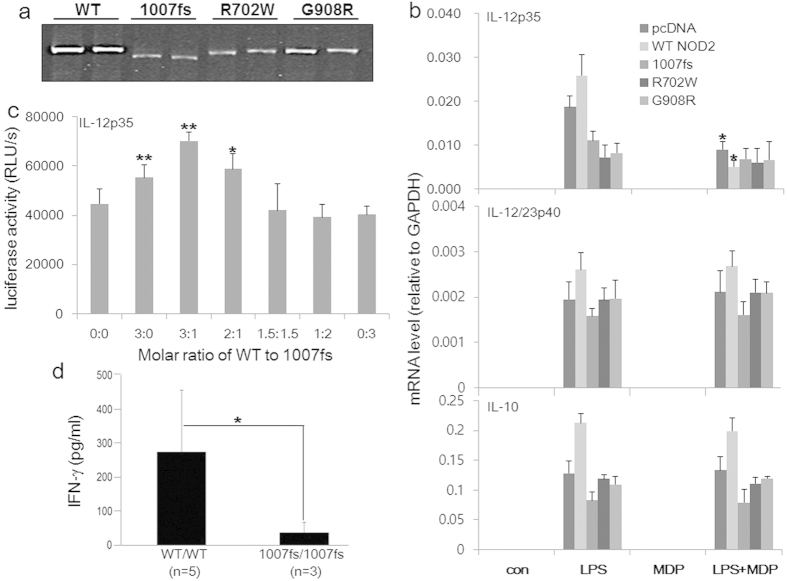
CD-associated NOD2 mutants selectively inhibit IL-12p35 transcription. (**a**) Western blot analysis of human NOD2 and its variants. The expression vectors contain a FLAG tag. Following transient transfection in duplicates, whole cell lysates were analyzed with a FLAG-specific mAb. Although cropping was used in the final image presentation for the purpose of saving space (the red-box defines the cropped boundary), all lanes were taken from the same gel in the original ordering (see Supplemental Fig. 1s for the original gel). (**b**) Human macrophage cell line THP-1 was transiently transfected with expression vectors for human NOD2 or its CD-associated variants as indicated. Cells were then stimulated with LPS (500 ng/ml) in the presence or absence of MDP (5 μg/ml) for 6 h, harvested and mRNA expression of IL-12p35, p40 and IL-10 analyzed by real time RT-PCR. pcDNA3 was used as an empty control vector. Data represent four independent experiments. (**c**) Luciferase activity in lysates of cells transfected with the human IL12p35 promoter-luciferase reporter, together with various molar ratios of vectors encoding NOD2 and 1007fs, then stimulated for 7 h with LPS before harvesting. Data are summary of 3 independent experiments. (**d**) IFN-γ production by human PBMCs from five healthy controls and three CD patients homozygous for 1007fs.

To further investigate the molecular mechanisms whereby the NOD2 mutations may impact on IL-12 gene expression we expressed recombinant NOD2 and the three major CD-associated mutants: 1007fs, R702W, and G908R. These mutants were expressed at mRNA and protein levels almost as efficiently as the WT NOD2 (Fig. 8a). In contrast to the enhancing activity of the low-dose NOD2, the three mutants inhibited p35 transcription in the absence of MDP (Fig. 8b, upper), but not p40 (middle), nor IL-10 (lower). Thus, it appears that the NOD2 mutants have an “acquired activity” selectively inhibiting p35 gene transcription in a MDP-independent manner.

To determine if NOD2 and 1007fs could functionally compete (for example, in a heterozygous person) we cotransfected NOD2 and 1007fs together at various molar ratios into RAW264.7 cells, together with the IL12p35 promoter-luciferase reporter. When 1007fs became more abundant than NOD2, it countered the stimulatory effect of NOD2 on p35 transcription (Fig. 8c). Since we have previously demonstrated that NOD2 and 1007fs could physically associate with one another^6^, it is very likely that 1007fs’ IL-12p35-inhibiting activity was the result from its “dominant negative” (DN) activity against NOD2 via physical association with each other.

These results are also largely consistent with a report that human DCs derived from CD patients bearing heterologous 1007fs mutation, when infected *in vitro* with the Gram-negative bacteria *Salmonella typhimurium*, were severely deficient in IL-12 production^40^. Our own analysis of three homozygous 1007fs-CD patients also revealed severely impaired IFN-γ production by their PBMCs stimulated *in vitro* with intact *Mycobacterium tuberculosis* (Fig. 8d), which implies an underlying defect in the production of IL-12, the most important inducer of IFN-γ production by activated T and NK cells.

## Discussion

A number of novel and potentially important findings have been obtained from this work. We have uncovered an unappreciated regulatory crosstalk between the TLR4- and NOD2-mediated signaling pathways of NOD2, being able to regulate the expression of a large number of genes by LPS-activated macrophages. The comprehensive RNAseq analyses of the global gene expression comparisons between WT and NOD2-deficient macrophages have clearly revealed the cross-regulatory nature of the TLR4- and NOD2-mediated signaling in macrophage activation. The probing also specifically identified GDF-15 as the most downregulated gene in the absence of NOD2. GDF15 is a member of the TGF-β superfamily^41^, which is widely distributed in mammalian tissues and has been shown to play multiple roles in various pathologies, including inflammation, cancer, cardiovascular diseases, and obesity^42^. GDF-15 serum levels are a highly reliable predictor of disease progression. However, the biological significance underlying these observations is far from clear. GDF15 could have a positive or negative role depending on the state of cells or their environment^43^. We speculate that GDF15 may be a crucial anti-inflammatory factor limiting macrophage activation in a NOD2-dependent manner. It is noteworthy that the majority of the large numbers of genes strongly upregulated in the absence of NOD2 represent proteolytic enzymes and peptidases involved in inflammation, suggesting that the principle way by which NOD2 limits tissue damages by activated macrophages is to control the production of these enzymes during inflammatory responses.

Further, we have identified hematopoietically derived C/EBPα as a critical balancing regulator between host defense and inflammation. This crosstalk results in balanced regulation of IL-12 production depending on the inflammatory intensities of the cell. To our knowledge, this is the first study demonstrating a potential role of C/EBPα in CD. Previously, genome-wide association studies have identified protein tyrosine phosphatase, non-receptor type 2 (PTPN2) as susceptibility gene for IBD^44^,^45^,^46^. Interestingly, a recent in silico analysis revealed that the CD-associated *PTPN2* SNP rs7234029 modulates potentially the binding sites of several transcription factors involved in inflammation including GATA-3, NF-κB, C/EBP, and E4BP4^47^.

TNBS colitis exhibits heightened Th1-Th17 response (increased IFN-γ and IL-17) as the disease becomes chronic, similarly to human CD^6^,^48^,^49^,^50^. Our data here lends support to the notion that overexpression of IL-12, not IL-23, is responsible for exacerbated intestinal inflammation in the acute disease model as a result of the loss of hematopoietic C/EBPα-mediated regulation. It should be noted that a study by Biswas *et al.* in NOD2-deficient mice inoculated with *Helicobacter hepaticus* indicated that non-hematopoietic NOD2 may play a more important role in this model^51^. This finding exemplifies the complex and controversial nature of studies of NOD2’s broad biological activities in the regulation of host defense mechanisms and intestinal inflammation that involve the use of many different models focusing on various aspects and anatomic locations of the disease, genetic background, housing conditions, and disease triggers.

Thirdly, we further elucidated how the CD-associated human mutations in NOD2, 1007fs in particular, may act as “dominant negatives” to counter the normal activities of NOD2. These mutants, by virtue of their inability to bind MDP, lose the activity to induce NF-κB, thus the production of inflammatory cytokines and host responses to invading pathogens (“loss of function”). What we have uncovered in this study is a “gain of function” activity of these mutants to selectively inhibit LPS-induced IL-12 production in an MDP-independent manner by acting as “dominant negatives” against NOD2 via physical association with each other. This finding provides a better explanation than the current dogmas for how the disease develops in patients bearing these mutations, a highly controversial issue that has been dogging the field of CD research for years. Our results are largely consistent with the report that human DCs derived from CD patients bearing heterologous 1007fs mutation, when infected *in vitro* with the Gram-negative bacteria *Salmonella typhimurium*, were severely deficient in IL-12 production^40^. This notion is also supported experimentally by a study of Morosky *et al.* showing that NOD2 negatively regulates type I interferon induction by retinoid acid-induced gene-I (RIG-I) in inflammatory signaling, and that the three main NOD2 mutants form an interaction with RIG-I and negatively regulate its signaling to a greater extent than WT NOD2^52^. The “dominant negative” role of 1007fs has also been observed in cells infected with human cytomegalovirus in which virus-induced NOD2 signaling initiates innate immune responses and restricts virus replication^53^.

A significant implication of the observed “acquired” and “intrinsic” activity of 1007fs to inhibit IL-12 production selectively is that in 1007fs-heterozygous patients, the mutation may lead to diminished host defense against pathogens when the activating signal MDP is not present, whereas its inability to respond to MDP (“loss of function”) would result in exacerbated inflammation in the face of mixed infectious agents producing both LPS and MDP. In CD patients carrying NOD2 mutations on the other hand, a principally immunodeficient phenotype is predicted and supported by clinical observations that humoral or cellular immune defects caused by impaired cytokine production can predispose to exacerbated intestinal mucosal inflammation^54^. The immunodeficiency theory further hypothesizes that defects in innate immunity leading to compensatory immune processes underlie the CD phenotype and suggests that therapy should stimulate immunity rather than suppress it^55^. Indeed, therapeutic approaches such as the clinical use of antibiotic therapy or granulocyte macrophage colony stimulating factor are consistent with the concept of an immunodeficiency being a crucial element in CD.

In summary, our study demonstrates that the signaling pathways of TLR4 and NOD2 can mutually regulate one another in inflammatory cells. NOD2 signaling activated by MDP, in a RIP2-dependent manner, induces the phosphorylation of C/EBPα via PKC at serine 248. Activated C/EBPα then binds to the IL-12p35 promoter at the “AAAAATGTGG” sequence between –31 and –22, blocking its transcriptional initiation. The precise role of RIP2 in this pathway remains to be further elucidated. Modulation of IL-12 production serves to maintain intestinal immune balance and tissue homeostasis via an elaborate network of players. Disturbance in the crosstalk between these two pathways, such as those caused by the disease-associated NOD2 mutations, may shift the homeostatic balance in a microbial context-dependent manner. To restore the impaired homeostasis would be a way forward to developing novel therapeutic strategies.

## Methods

### Cells

The murine macrophage-like cell line, RAW264.7, was obtained from American Type Culture Collection (Manassas, VA) and maintained in RPMI1640 containing 10% FBS, 2 mM L-glutamine, and penicillin/streptomycin. Bone marrow derived macrophages were isolated from mice and induced the differentiation for 7 day in DMEM containing 10% FBS, 20% L929 conditioned medium and penicillin/streptomycin. Medium was changed twice a week during maintaining.

### Reagents and Plasmids

Recombinant murine and human IFN-γ were purchased from R&D system. LPS from *E. coli* 0127:B8 was from Sigma-Aldrich. Bay11–7082 was kindly provided by Dr. E. Cesarman of Weill Cornell Medical College, staurosporine was from Calbiochem (Madison, WI). The wild type (WT) C/EBPα expression vector and its mutant (S21A, T222A/T226A, S193A, and S248A) expression vectors were generously provided by Dr. MacDougald (University of Michigan Medical School, Ann Arbor, MI). The human Nod2 expression vector and the 3020insC Nod2 mutant were generated by overlapping PCR, were reported previous our report^6^. Anti-mouse IL-12 antibody (C17.8), and its control antibody was from BioXcell. Anti-mouse IL-23 (G23-8) and its control antibody were from eBioscience.

### Mice

Nod2-knockout (KO), C/EBPα-conditional KO and control mice were purchased from The Jackson Laboratories (Bar Harbor, ME). C/EBPα-conditional KO mice were homozygous for C/EBPα floxed allele and hemizygous for the Mx1-Cre transgene. To induce Mx1-Cre gene expression, 3 weeks old mice were injected i.p. with 250 μg of polyinosinic-polycytidylic acid [poly(I:C)] (Sigma Aldrich, St. Louis, MO), every other day for 2 weeks as we previously reported^36^. All animal procedures were conducted in accordance with the institutional IACUC’s approved guidelines of Shanghai Jiaotong University and Weill Cornell Medical College.

### Induction of colitis

Conditional deletion of C/EBPα using poly I-C was performed as we described previously^36^. For colitis induction, TNBS (3.5 mg) in 100 μl of 45% ethanol was injected intrarectally into mice on day 0. MDP (100 μg) or PBS was injected on days –3, –2, and –1 via i.p., relative to TNBS injection. Neutralizing antibodies (250 μg) were injected i.p. on days 0 and 2.

### Histological analysis

Colon tissues were harvested on day 4. They were stained with H&E and analyzed for inflammation scores on a scale of 1–5, as described by Watanabe *et al.*^13^. Histology was scored as follows: 0, no sign of inflammation; 1, very low level; 2, low level of leukocytic infiltration; 3, high level of leukocytic infiltration, high vascular density, and thickening of the colon wall; and 4, transmural infiltrations, loss of goblet cells, high vascular density, and thickening of the colon wall.

### Electroporation and luciferase assay

Transient transfection in RAW264.7 cells was performed by electroporation as previously described (ref). Amaxa Cell Line Nuclerofector Kit V was used for transient transfection in THP1 cells as following manufacture’s protocol. Luciferase assay was performed as previously described^23^.

### Cytokine assays

mIL-12p40, mIL-12p70, mIL-10, and mTNF-a cytokine secretion was measured by the respective ELISA kits from BD Pharmingen (San Diego, CA). Mouse IL-23 was measured by an ELISA kit from Biolegend (San Diego, CA). All experiments were performed three times independently.

### Quantitative real-time PCR

mRNA were isolated using the RNeasy mini kit (Qiagen, Hilden, Germany) and reverse-transcribed into cDNA. Real-time PCR was performed with an AB17400 System using the SYBR GREEN PCR kit (Applied Bioscience). The following primers were used for PCR amplification: 1) mouse IL-12/IL-23p40, 5′-GGAAGCACGGCAGCAGAATAAAT-3′, 5′-AACTTGAGGGAGAAGTAGGAATGG- 3′, 2) mouse IL-12p35, 5′-CCCTTGCCCTCCTAAACCAC, 5′-TAGTAGCCAGGCAACTCTCG-3′.

### Chromatin-immunoprecipitation assays (ChIP)

ChIP assay was performed as previously described^23^. The primers in the mouse IL-12p35 promoters: 5′-CACAGTCCTGGGAAAGTCCT −3′, 5′-CGGCCCTCAGGTACTTACAG -3′.

### RNA sequencing and data analysis

Total RNA was isolated from mouse bone marrow macrophages using the RNeasy mini kit (Qiagen) according to manufacturer’s instructions. The quantity and quality of total RNA were checked by Nanodrop 8000 (Thermo Scientific) and Bioanalyzer 2100 (Agilent). Sequencing libraries for whole transcriptome analysis were generated following illumine TruSeq RNA low sample preparation protocol. Libraries were quantified by using a 2100 BioAnalyzer(Agilent) and Qubit Fluorometer 2.0 (invitrogen). Nine pM denatured libraries were loaded on an illumine HiSeq 2500 instrument using single read clustering and 51 cycles sequencing (per lane). Program Tophat was used to align the sequenced reads to the UCSC mm9 mouse reference genome, and program Cufflinks was used to measure transcript abundances in reads per kilobase of exon model per million mapped reads (RPKM).

The authors confirm that all animal experimental protocols were approved by the IACUC committee of Shanghai Jiaotong University (SJTU), and all *in vitro* experiments adhered to the biosafety standards approved by the Environmental Health and Safety Committee of SJTU.

## Additional Information

**How to cite this article**: Kim, H. *et al.* A novel crosstalk between TLR4- and NOD2-mediated signaling in the regulation of intestinal inflammation. *Sci. Rep.* **5**, 12018; doi: 10.1038/srep12018 (2015).

## Supplementary Material

Supplementary information

## Figures and Tables

**Table 1:** Differentially expressed genes in LPS-activated WT and NOD2 KO macrophages.

Gene_id	Refseq_id	WT (+LPS)	NOD2 KO (+LPS)	Fold_change
Gdf15 (growth differentiation factor 15)	NM_011819	65.24	2.84	+23.0
BC100530 (unannotated)	NM_001082546	2.89	367.83	−127.1
Stfa1 (stefin A1)	NM_001082543	2.49	310.22	−124.7
Mpo (myeloperoxidase)	NM_010824	2.06	195.71	−95.2
Ctsg (cathepsin G)	NM_007800	2.48	220.09	−88.7
Stfa2 (stefin A2)	NM_001082545	1.63	135.43	−83.2
Ngp (neutrophilic granule protein)	NM_008694	8.17	578.86	−70.8
Ms4a3 (membrane-spanning 4-domains, subfamily A, member 3)	NM_133246	2.20	151.74	−69.1
Prtn3 (proteinase 3)	NM_011178	2.94	192.43	−65.4
Stfa2l1 (stefin A2 like 1)	NM_173869	2.33	139.49	−59.9
S100a9 (S100 calcium binding protein A9)	NM_009114	69.85	3561.00	−51.0
Pglyrp1 (peptidoglycan recognition protein 1)	NM_009402	1.43	66.34	−46.4
Ltf (lactotransferrin)	NM_008522	1.24	55.51	−44.6
Gm5483 (predicted gene 5483)	NM_001082547	8.52	367.49	−43.1
Camp (cathelicidin antimicrobial peptide)	NM_009921	4.49	130.54	−29.1
Ccnb1 (cyclin B1)	NM_172301	1.34	31.71	−23.7
Rrm2 (ribonucleotide reductase M2)	NM_009104	3.02	61.69	−20.4
Chi3l3 (chitinase-like 3)	NM_009892	18.92	372.37	−19.7
Gm5416 (predicted gene 5416)	NM_001082542	3.39	65.36	−19.3
Ifitm1 (interferon induced transmembrane protein 1)	NM_001112715, NM_026820	13.31	251.88	−18.9
Cenpa (centromere protein A)	NM_007681	1.11	20.85	−18.8
Ly6c2 (lymphocyte antigen 6 complex, locus C2)	NM_001099217	43.30	790.80	−18.3
Birc5 (baculoviral IAP repeat-containing 5)	NM_009689, NM_001012273	2.24	39.67	−17.7
Lmnb1 (lamin B1)	NM_010721	1.81	31.49	−17.4
Ube2c (ubiquitin-conjugating enzyme E2C)	NM_026785	3.01	51.79	−17.2
2810417H13Rik (RIKEN cDNA 2810417H13 gene)	NM_026515	3.39	56.80	−16.8
Cdca3 (cell division cycle associated 3)	NM_013538	1.28	21.25	−16.7
Phgdh (3-phosphoglycerate dehydrogenase)	NM_016966	2.59	41.79	−16.2
Prc1 (protein regulator of cytokinesis 1)	NM_145150	1.12	17.55	−15.7
Chil1 (chitinase-like 1)	NM_007695	2.78	42.21	−15.2
Nusap1(nucleolar and spindle associated protein 1)	NM_133851, NM_001042652	1.12	16.97	−15.2
Cdca8(cell division cycle associated 8)	NM_026560	1.92	25.78	−13.4
Top2a(topoisomerase (DNA) II alpha)	NM_011623	3.18	42.06	−13.2
Ccna2(cyclin A2)	NM_009828	2.10	27.53	−13.1
Serpinb1a(serine (or cysteine) peptidase inhibitor, clade B, member 1a)	NM_025429	2.19	21.06	−9.6
Uhrf1 (ubiquitin-like, containing PHD and RING finger domains, 1)	NM_001111078, NM_001111080, NM_001111079, NM_010931	1.42	13.36	−9.4
Tk1 (thymidine kinase 1)	NM_009387, NM_001271729	1.31	12.29	−9.4
Hells (helicase, lymphoid specific)	NM_008234	1.42	13.37	−9.4
Stfa3 (stefin A3)	NM_025288	92.96	860.86	−9.3
Tacc3 (transforming, acidic coiled-coil containing protein 3)	NM_001040435	2.61	24.05	−9.2
Rad51 (RAD51 homolog)	NM_011234	1.50	13.76	−9.2
Rab27a (RAB27A, member RAS oncogene family)	NM_023635	1.12	10.13	−9.0
Prim1(DNA primase, p49 subunit)	NM_008921	1.27	11.48	−9.0
Mcm10(minichromosome maintenance deficient 10)	NM_027290	1.56	14.01	−9.0
Rrm1(ribonucleotide reductase M1)	NM_009103	3.99	35.64	−8.9
Zwilch(zwilch kinetochore protein)	NM_026507	1.00	8.77	−8.7
2010005H15Rik(RIKEN cDNA 2010005H15 gene)	NM_029733	13.79	120.04	−8.7
Smc2(structural maintenance of chromosomes 2)	NM_008017	2.23	19.11	−8.6
Cks2(CDC28 protein kinase regulatory subunit 2)	NM_025415	6.73	55.78	−8.3
Sell(selectin, lymphocyte)	NM_001164059, NM_011346	9.07	74.18	−8.2
Gm15056(predicted gene 15056)	NM_001177471	1.47	11.98	−8.2
Uck2(uridine-cytidine kinase 2)	NM_030724	2.31	18.62	−8.1
Lbr(lamin B receptor)	NM_133815	1.77	13.97	−7.9
Rfc4(replication factor C (activator 1) 4)	NM_145480	1.34	10.34	−7.7
Tipin(timeless interacting protein)	NM_025372	2.76	20.83	−7.6
Cdk1(cyclin-dependent kinase 1)	NM_007659	3.02	22.83	−7.6
Oip5(Opa interacting protein 5)	NM_001042653	1.30	9.79	−7.5
Cks1b(CDC28 protein kinase 1b)	NM_016904	5.54	41.58	−7.5
1100001G20Rik(WAP four-disulfide core domain 21)	NM_183249	55.58	401.58	−7.2
Hmgn2(high mobility group nucleosomal binding domain 2)	NM_016957	38.26	275.98	−7.2
Mcm5(minichromosome maintenance deficient 5, cell division cycle 46)	NM_008566	3.49	24.77	−7.1
Mcm2(minichromosome maintenance deficient 2 mitotin)	NM_008564	2.68	18.69	−7.0
Ltb4r1(leukotriene B4 receptor 1)	NM_008519	1.26	8.76	−6.9
Lipg(lipase, endothelial)	NM_010720	3.34	23.00	−6.9
Fignl1(fidgetin-like 1)	NM_021891, NM_001163360, NM_001163359	1.80	12.10	−6.7
Cenpe(centromere protein E)	NM_173762	1.36	8.86	−6.5
Asf1b(anti-silencing function 1B histone chaperone)	NM_024184	2.27	14.70	−6.5
Mcm3(minichromosome maintenance deficient 3)	NM_008563	5.19	33.55	−6.5
Gins1(GINS complex subunit 1 (Psf1 homolog))	NM_001163476, NM_027014	1.65	10.61	−6.4
Mcm4(minichromosome maintenance deficient 4 homolog)	NM_008565	3.03	19.02	−6.3
Arhgap19(Rho GTPase activating protein 19)	NM_001163495, NM_027667	1.18	7.22	−6.1

Differentially expressed genes in WT and NOD2 KO macrophages treated with LPS. Bone marrow macrophages from WT and NOD2 KO mice were stimulated with LPS with or without MDP. Total RNA were extracted for RNA-seq analysis. Criteria for selection of genes: minimum expression level: 1; minimum fold change: 6.0, which is the threshold level that can be reliably corroborated by real time RT-PCR. Positive values represent expression ratios of WT/KO whereas negative values represent KO/WT ratios. Criteria for selection of genes: minimum expression level: 1; minimum fold change: 6.0, which is the threshold level that can be reliably corroborated by real time RT-PCR. Positive values represent expression ratios of WT/KO whereas negative values represent KO/WT ratios.

**Table 2:** Genes differentially expressed in WT vs NOD2 KO macrophages treated with MDP and LPS.

		Expression Level		
Gene_id/Gene_name	Refseq_id	WT (LPS+MDP)	NOD2 KO (LPS+MDP)	Fold_change
Gdf15 (growth differentiation factor 15)	NM_011819	60.00	3.74	+16.0
H2-M2 (histocompatibility 2, M region locus 2)	NM_008204	36.32	2.96	+12.3
Sdc1 (syndecan 1)	NM_011519	29.50	4.15	+7.1
H2-L (histocompatibility 2, D region locus L)	NM_001267808	198.33	29.70	+6.7
Igfbp7 (insulin-like growth factor binding protein 7)	NM_001159518, NM_008048	7.22	1.21	+6.0
Ngp (neutrophilic granule protein)	NM_008694	1.68	672.15	−399.4
Stfa1 (stefin A1)	NM_001082543	1.77	400.15	−226.2
BC100530 (stefin A-like protein)	NM_001082546	2.66	498.98	−187.6
Prtn3 (proteinase 3)	NM_011178	1.20	210.26	−175.3
Stfa2l1 (stefin A2 like 1)	NM_173869	1.39	193.70	−139.5
S100a9 (S100 calcium binding protein A9, or calgranulin B)	NM_009114	30.74	3973.45	−129.2
Stfa2 (stefin A2)	NM_001082545	1.43	174.03	−121.5
2810417H13Rik	NM_026515	1.36	58.47	−42.9
Rrm2 (ribonucleotide reductase M2)	NM_009104	1.49	59.87	−40.1
Birc5 (baculoviral IAP repeat- containing 5)	NM_009689, NM_001012273	1.15	42.02	−36.5
Ube2c (ubiquitin-conjugating enzyme E2C)	NM_026785	1.50	52.80	−35.2
Phgdh (3-phosphoglycerate dehydrogenase)	NM_016966	1.62	46.49	−28.7
Chi3l3 (chitinase-like 3)	NM_009892	15.31	424.85	−27.8
Top2a (topoisomerase II alpha)	NM_011623	1.82	42.86	−23.5
Ifitm1 (interferon induced transmembrane protein 1)	NM_001112715, NM_026820	11.54	263.59	−22.9
Sell (selectin, lymphocyte)	NM_001164059, NM_011346	3.57	77.47	−21.7
Camp (cathelicidin antimicrobial peptide)	NM_009921	7.68	160.69	−20.9
Plac8 (placenta-specific 8)	NM_139198	58.68	1166.53	−19.9
Stmn1 (stathmin 1)	NM_019641	4.29	85.24	−19.9
Hmgb2 (high mobility group box 2)	NM_008252	5.43	100.04	−18.4
Ccna2 (cyclin A2)	NM_009828	1.69	28.79	−17.1
Lmnb1 (lamin B1)	NM_010721	2.12	32.74	−15.4
Mcm7 (minichromosome maintenance deficient 7)	NM_008568	1.82	26.94	−14.8
Chi3l1 (chitinase-like 1)	NM_007695	3.16	45.36	−14.4
Uhrf1 (ubiquitin-like, containing PHD and RING finger domains, 1)	NM_001111078, NM_001111080, NM_001111079, NM_010931	1.01	13.77	−13.6
Gm5483 (predicted gene 5483)	NM_001082547	33.08	444.33	−13.4
Ly6c2 (lymphocyte antigen 6 complex, locus C2)	NM_001099217	63.70	854.05	−13.4
Cdca8 (cell division cycle associated 8)	NM_026560	2.03	26.12	−12.9
Rrm1 (ribonucleotide reductase M1)	NM_009103	2.99	35.24	−11.8
Mcm2 (minichromosome maintenance deficient 2)	NM_008564	1.59	18.25	−11.5
Ckap2l (cytoskeleton associated protein 2-like)	NM_181589	1.15	13.06	−11.3
Smc2 (structural maintenance of chromosomes 2)	NM_008017	1.78	19.73	−11.1
Fignl1 (fidgetin-like 1)	NM_021891, NM_001163360, NM_001163359	1.05	11.65	−11.1
Tacc3 (transforming, acidic coiled-coil containing protein 3)	NM_001040435	2.22	23.91	−10.8
Mcm5 (minichromosome maintenance deficient 5)	NM_008566	2.27	23.49	−10.4
Cdk1 (cyclin-dependent kinase 1)	NM_007659	2.51	25.52	−10.2
Hmgn2 (high mobility group nucleosomal binding domain 2)	NM_016957	30.41	291.25	−9.6
Serpinb1a (serine peptidase inhibitor, clade B, member 1a)	NM_025429	2.27	20.99	−9.2
Rfc4 (replication factor C4)	NM_145480	1.05	9.48	−9.0
Igfbp4 (insulin-like growth factor binding protein 4)	NM_010517	3.47	31.14	−9.0
Ly6c1 (lymphocyte antigen 6 complex, locus C1)	NM_010741, NM_001252058, NM_001252055, NM_001252057, NM_001252056	1.88	16.49	−8.8
Rab27a (RAB27A, member RAS oncogene family)	NM_023635	1.35	11.57	−8.6
Gmnn (geminin)	NM_020567	3.27	27.03	−8.3
Gm5416 (predicted gene 5416)	NM_001082542	9.72	79.34	−8.2
Cks1b (CDC28 protein kinase 1b)	NM_016904	5.61	45.08	−8.0
Ltb4r1 (leukotriene B4 receptor 1)	NM_008519	1.24	9.80	−7.9
Mcm3 (minichromosome maintenance deficient 3)	NM_008563	4.20	33.30	−7.9
Lbr (lamin B receptor)	NM_133815	1.89	14.78	−7.8
Myc (myelocytomatosis oncogene)	NM_001177354, NM_001177352, NM_001177353, NM_010849	1.88	14.08	−7.5
Tpx2 (TPX2, microtubule-associated protein homolog)	NM_001141977, NM_001141976, NM_001141975, NM_028109, NM_001141978	2.51	18.81	−7.5
Mcm4 (minichromosome maintenance deficient 4)	NM_008565	2.52	18.65	−7.4
Tipin (timeless interacting protein)	NM_025372	3.01	21.31	−7.1
Asf1b (anti-silencing function 1B histone chaperone)	NM_024184	2.07	14.57	−7.1
Gpr97 (G protein-coupled receptor 97)	NM_173036	1.06	7.45	−7.0
Lig1 (ligase I, DNA, ATP- dependent)	NM_001199310, NM_010715, NM_001083188	2.38	16.43	−6.9
Il18rap (interleukin 18 receptor accessory protein)	NM_010553	1.29	8.70	−6.8
Gins1 (GINS complex subunit 1 (Psf1 homolog))	NM_001163476,NM_027014	1.43	9.62	−6.8
Tuba8 tubulin, alpha 8	NM_017379	2.24	14.99	−6.7
Trip13 (thyroid hormone receptor interactor 13)	NM_027182	1.74	11.61	−6.7
Lsm5 (LSM5 homolog, U6 small nuclear RNA associated)	NM_025520	7.97	52.46	−6.6
Atad2 (ATPase family, AAA domain containing 2)	NM_027435	2.05	13.01	−6.4
Syce2 (synaptonemal complex central element protein 2)	NM_001168244, NM_027954, NM_001168246, NR_031759	2.90	18.04	−6.2
Dkc1 (dyskeratosis congenita 1, dyskerin	NM_001030307	1.28	7.96	−6.2
Hsh2d (hematopoietic SH2 domain containing)	NM_197944	1.85	11.31	−6.1
Haus4 (HAUS augmin-like complex, subunit 4)	NM_145462	1.16	6.96	−6.0
S100a8 (S100 calcium binding protein A8 (calgranulin A)	NM_013650	799.33	4789.38	−6.0
Cenpv (centromere protein V)	NM_028448	1.14	6.83	−6.0
Arhgap15 (Rho GTPase activating protein 15)	NM_153820, NM_001025377	1.76	10.52	−6.0

Differentially expressed genes in WT vs NOD2 KO macrophages treated with MDP and LPS. Criteria for selection of genes are the same as described in Table 1. Positive values represent expression ratios of WT/KO whereas negative values represent KO/WT ratios. Criteria for selection of genes: minimum expression level: 1; minimum fold change: 6.0, which is the threshold level that can be reliably corroborated by real time RT-PCR. Positive values represent expression ratios of WT/KO whereas negative values represent KO/WT ratios.
