# Supplementary material for: A novel crosstalk between TLR4- and NOD2-mediated signaling in the regulation of intestinal inflammation
Source: Sci Rep. 2015 Jul 8;5:12018. doi: 10.1038/srep12018 (PMC4495563; doi:10.1038/srep12018)
Supplement: Supplementary information [file srep12018-s1.pdf]

A novel crosstalk between TLR4- and NOD2-mediated signaling in the regulation of intestinal inflammation

Hajeong Kim[1], Quanju Zhao[1,2], Hua Zheng[1], Xin Li[2,3], Tuo Zhang[2], Xiaojing Ma[1,2]

1. State Key Laboratory of Microbial Metabolism, Sheng Yushou Center of Cell Biology and Immunology and School of Life Sciences & Biotechnology, Shanghai Jiao Tong University 800 Dongchuan Road, Shanghai, China 200240
2. Department of Microbiology and Immunology, Weill Cornell Medical College, 1300 York Avenue, New York, NY, USA 10065
3. Department of Breast Surgery, Xiangya Hospital, Central South University, 87 Xiangya Road, Changsha, Hunan, China 410008

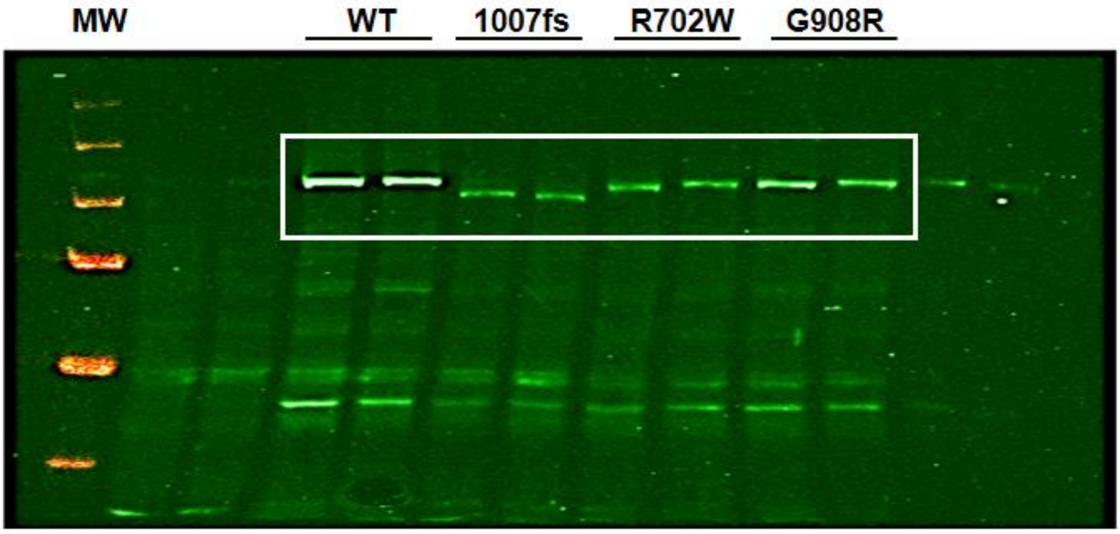

Supplemental Fig 1s: Original gel of Fig 8a. The white-boxed area is the cropped image shown in Fig 8a.
